# Evolving Spinal Treatment Modalities: A Review of the Literature on Non-surgical Interferential Differential Dynamics (IDD) Therapy

**DOI:** 10.7759/cureus.59873

**Published:** 2024-05-08

**Authors:** Anuj Gupta

**Affiliations:** 1 Orthopedics: Spine, Triveni Ortho and Spine Center, Delhi, IND; 2 Spine, Max Superspeciality Hospital, Delhi, IND

**Keywords:** deep oscillation therapy, conservative therapy, spinal decompression, idd, low-back pain (lbp)

## Abstract

Low back pain is one of the most common ailments encountered by physicians and orthopedic surgeons. There are various modalities used to treat low back pain, including conservative management, and a few of them involve rest, medications, massage, bracing, acupuncture, and physical therapy. Though most of the patients improve with conservative management, the burden of this disease has been very high and caused a significant amount of economic loss. Therefore, in-depth knowledge of all conservative methods is essential for physicians managing low back pain. Furthermore, there can be many causes of low back pain. Some of the more common ones are mechanical back pain due to paraspinal muscles or facetal in origin, discogenic back pain, and sacroiliac joint dysfunction. Many patients, especially the older population, have the discogenic origin as the more common cause of back pain, and traction therapy has been used for its treatment for ages. In this review, we discuss non-surgical spinal decompression/traction therapy popularly known as interferential differential dynamics (IDD) therapy with its current standing and recent advancement.

## Introduction and background

Low back pain (LBP) is one of the most common ailments and almost 80-90% of men and women suffer from LBP once in their lifetime [1]. There can be multiple aetiologies for LBP. It can involve musculature, ligaments, vertebral bodies, and intervertebral discs. It has been observed that intervertebral disc issues are responsible for more than 80% of cases presenting with low back pain [2]. Despite such a high prevalence, the percentage of patients requiring surgical management for LBP is very low [3]. Therefore, it can be implied from this information that conservative management is the best management option for most patients with LBP. The absolute indications for surgery include intractable LBP, radicular pain, weakness in lower limbs, and cauda equina syndrome due to severe nerve compression.

Conservative management for LBP offers a broad choice of different modalities within different specialties, based on the clinicians’ experience and training. Modalities included in conservative management are rest, medications, massage, bracing, acupuncture, and physical therapy. Physical therapy could include heat, exercises, traction, decompression (an advanced form of traction), or spinal manipulation [4].

The role of traction in LBP is time immemorial. There are many published studies in the existing literature that mention the effects of traction [5-7]. Some published studies are not convinced of the benefits of traction and call it a sham therapy [8,9]. However, in recent years, there have been advancements in traction-based therapies due to a better understanding of disc dynamics and the mechanism of disease. Additionally, technological advancements with computerized algorithms have improved how mechanical forces can regulate biological activity.

We intend to review the literature and understand the mechanism, benefits, and current standing of advanced modalities rooted in traction, particularly those with the added intended use of decompression, in cases of LBP.

## Review

To evaluate more about decompression therapy in LBP, we reviewed many published articles, including case reports, review articles, case series, and randomized studies. In particular, we considered the IDD therapy treatment device available commercially with the name Accu-SPINA by North American Medical Corporation (Marietta, GA, United States). Early papers often referred to the IDD therapy treatment as “traction”; however, its 510K clearance describes one of the first decompression labeling claims permitted by the FDA.

We evaluated 14 studies related to intervertebral differential dynamics (IDD) therapy. We tried to find out the mechanism of IDD therapy, its uses, and its current status.

LBP is one of the most common problems in developing and developed countries. It is estimated that there is an economic loss of more than 100 billion dollars annually in the USA [10,11]. Even though it is such a common disease, most patients respond to conservative management. The indications of surgical management are limited to neurological deficits and intractable pain [12]. There are different modalities of conservative management, which include prescribing medication, transforaminal injection [13], massage, bracing, and physical therapy. Every physician has a different means or combination of administering modalities for treating patients depending on their subjective experience. One of the most commonly used modalities was conventional traction.

The different modes of traction are manual spinal traction, mechanical traction, non-surgical decompression, and IDD therapy. While traction modalities have been broadly utilized in physical medicine, a review of early literature, using PubMed, on non-surgical decompression, indicated that published studies had not identified a clear distinction between conventional traction as compared to decompression or decompression with specific force applications or oscillation. It has been known that conventional traction increases the disc pressure due to the contraction of back muscles [14]. Thereafter, various devices have been introduced offering technological solutions aimed at eliminating the drawbacks seen with conventional traction for LBP patients. One such device began as a decompression modality in early 2000 and was later enhanced to employ more advanced algorithms only possible with the advent of higher-speed processors. The IDD therapy concept draws from the field of mechanobiology to deliver highly specific, differentiating treatment forces.

The mechanism of action in IDD therapy utilizes an oscillating, sinusoidal dynamic force movement intended to distract the disc space and free up the nerve roots and other epidural structures. Under imaging, rehydration of the disc material is seen along with an increase in blood supply which encourages the natural healing of the disc. The same has been confirmed by Guehring T et al. [15]. They did a molecular study on animal vertebrae where they found evidence of disc rehydration on MRI and histological examination of disc material after 28 days of distraction. Even in a cadaveric study by Gay RE et al., they found that the distraction forces reduce pressure in the nucleus pulposus [16]. However, the amount of reduction in pressure can have a direct correlation with the amount of degeneration of the disc before starting the decompressive distraction treatment.

IDD therapy is also known as an advanced form of non-surgical spinal decompression because it is the only decompression method on the market with technological innovations that were proven patent-worthy by the United States Patent and Trademark Office (USPTO). How is IDD therapy different? IDD therapy's precise angle mechanism can generate separate distraction forces at every level of lumbar intervertebral disc space. Therefore, it is more targeted as compared to conventional traction therapy. It promotes the diffusion of oxygen, water, and nutrients and, in turn, promotes rehydration. It has been shown that 25 sessions of treatment (20 sessions over 10 weeks and 5 maintenance sessions over 5 months) for 25-30 minutes each give the best results in most of the patients [17]. Cholewicki et al. found in their study minimal activity in truncal muscles after using IDD therapy and supports rehydration theory for the improvement seen in patients undergoing this therapy [18]. There are multiple studies published over the years that showed positive effects of IDD therapy over conventional traction therapy in patients with LBP and cervical pain as well [19-22]. Amjad F et al. conducted a randomized study where one group was given non-surgical decompression along with physical therapy and the other group was given only physical therapy [23]. They found that the combination of non-surgical decompression along physical therapy is clinically and statistically more effective than physical therapy alone.

Another advancement to non-surgical decompression with the IDD therapy treatment algorithm is the sinusoidal oscillations deliverable at each intervertebral disc level. This is apart from the primary distraction force that supports better rehydration and healing of the disc through better entry of oxygen, nutrients, and water. This effect of IDD therapy on the Accu-SPINA has been confirmed by videofluoroscopy by Busch RE et al. [24].

A randomized study by Schaufele demonstrates a similar effect of IDD as physical therapy when comparing changes in functional and pain scores in both study groups [25]. This study was focused on degenerative disc patients for whom the standard of care is typically exercise-based physical therapy. Despite an inadequate application of IDD (only 8 sessions in 6 weeks, or 32% of the recommended therapeutic protocol) the IDD group experienced pain relief and mobility equal to that of the physical therapy control group at the end of the study. Similarly, we found another randomized study by Schimmel JJ et al., which had several shortcomings (Table 1) [26]. First, there was a small sample size (31 vs 29). Second, the patients recruited in the study were not clinically established cases of LBP due to disc degeneration or herniation. The study mentions that an orthopedic surgeon reviewed the file of the patients along with X-rays, not MRI scans. As established by many studies, disc herniation can be asymptomatic in a large number of populations. Several causes of LBP need to be clinically segregated. Therefore, in this study, the origin of LBP was not clearly defined. Also, in the control group, the treatment was administered but not increased, which created a serious bias in the study. As the outcome was based on functional scores (visual analog scale (VAS), Oswestry disability index (ODI), 36-item short form survey (SF-36)), the responses of control group patients are not reliable, as they might be aware of the group they fall into. Lastly, perhaps the most significant study flaw was the researchers' failure to isolate a control group, as both groups were treated with IDD therapy. The researchers presumed that utilization of 10% or less of the patient’s body weight during treatment would constitute a non-therapeutic regimen. This may have, in effect, rendered the “sham” group a low-intensity IDD treatment group, not a true control group. Essentially then, all patients in the study received either a full-intensity or light-intensity regimen of IDD therapy treatment forces without a third critical control group, which should have received no IDD therapy treatment forces whatsoever. The researchers' true finding was that there was no difference between full-intensity IDD therapy (1/2 body weight + 10 lbs) versus low-intensity IDD therapy (10 lbs or less). Both groups reported a significant improvement in LBP, leg pain, ODI, and SF-36, and a decrease in the use of pain medication was reported in both groups.

**Table 1:** Some published studies evaluating the results of IDD therapy in LBP IDD: interferential differential dynamics; LBP: low back pain

Some of the reviewed studies			
Busch RE et al [24]	A single session of spinal decompression with oscillation and videofluoroscopy	Case report	2023
Amjad F et al [23]	Effects of non-surgical decompression therapy in addition to routine physical therapy on pain, range of motion, endurance, functional disability and quality of life versus routine physical therapy alone in patients with lumbar radiculopathy; a randomized controlled trial	RCT	2022
Šedienė A [27]	The effect of spinal stabilization exercises and intervertebral differential dynamics therapy on back pain and function condition in lumbar disc herniation	Case series	2022
Ekediegwu E et al [21]	Reduction in chronic low back pain using intervertebral differential dynamics therapy (IDDT) and routine physiotherapy: a retrospective pre-post study	Retrospective study	2021
Li J et al. [28]	The first non-surgical treatment of cervical and lumbar diseases in Guangxi - non-surgical spinal decompression system (IDD)	Case report	2021
Ekediegwu EC et al [22]	A case series of non-surgical spinal decompression as an adjunct to routine physiotherapy management of patients with chronic mechanical low back pain	Case series	2019
Patnaik G [17]	Role of IDD therapy in the back and neck pain	Review article	2018
Henry L [19]	Non-surgical spinal decompression an effective physiotherapy modality for neck and back pain	Research Article	2017
Kang JI et al [20]	Effect of spinal decompression on the lumbar muscle activity and disk height in patients with herniated intervertebral disk	Case series	2016
Schaufele MK [25]	Intervertebral differential dynamics (IDD) therapy vs. exercise based physical therapy –Results from a randomized controlled trial	RCT	2010
Gay RE et al [16]	Stress in lumbar intervertebral discs during distraction: a cadaveric study	Cadaveric Study	2008
Cholewicki J et al [18]	Trunk muscle response to various protocols of lumbar traction	Original Article	2009
Guehring T et al [15]	Disc distraction shows evidence of regenerative potential in degenerated intervertebral discs as evaluated by protein expression, magnetic resonance imaging, and messenger ribonucleic acid expression analysis.	Basic Science	2005
Schimmel JJ et al [26]	No effect of traction in patients with low back pain: a single centre, single blind, randomized controlled trial of intervertebral differential dynamics therapy	RCT	2009

## Conclusions

In most of the cases of LBP, conservative management is helpful. Appropriate knowledge of all forms of conservative management should be sought to effectively treat the patient and reduce the burden of the disease. The traction therapy has been a useful tool for treating LBP for ages and various modifications with time have made it better. The IDD therapy has promising outcomes in cases of LBP. There are several published studies in the literature in favor of IDD therapy. The inclusion of sinusoidal oscillations in IDD therapy utilizing the Accu-SPINA (North American Medical Corporation) has improved it further. More long-term studies with large sample sizes and long follow-ups are required to better establish this as a standard of care for LBP.
